# What Is Taught?

**Published:** 1887-05

**Authors:** P. P. Wells

**Affiliations:** Brooklyn, N. Y.


### HOMŒOPATHIC PHYSICIAN,

A MONTHLY JOURNAL OF MEDICAL SCIENCE.

"If our school ever gives up the strict inductive method of Hahnemann, we are lost, and deserve only to be mentioned as a caricature in the history of medicine."—CONSTANTINE HERING.

Vol. VII. MAY, 1887. No. 5.

### WHAT IS TAUGHT?

P. P. WELLS, M. D., BROOKLYN, N. Y.

We have on former occasions noticed the neglect and omissions by our colleges to teach their classes the knowledge necessary to make them the homœopathic physicians they have by the title of their institutions and their annual announcements promised. Our attention has been called to this by the testimony of their graduates and by the sorry evidence these have given of total absence of all knowledge of the philosophy and rules of practice which their diplomas falsely declare they have been taught, and that when leaving these graduating schools they were in such possession of this as to make them so capable of the practice of specific medicine as to be worthy of the confidence of the sick in the community, as specific, *i. e.*, homœopathic, healers. When so graduated and thus commended, we have found them wholly ignorant of the knowledge they were so declared to possess, and willing witnesses to this other damning fact, that there had been on the part of their teachers no attempt to impart this! Were we mistaken if we judged this state of things called for sharp rebuke? Were we mistaken when we deemed that the public should know of this neglect, and of the character of that which medical college faculties were giving their pupils as a substitute for that which had been promised?

These questions are suggested by statements of a member of one of our county medical societies to the effect that my last paper on "Medical Education" was denounced at their February meeting in a paper by one of its members, the object of which was to "*urge on our colleges that their professors teach pure Homœopathy.*" The reason for this denunciation was that my paper "*was too severe.*" This denunciation was approved of by another member (himself a member of one of these guilty faculties), and especially that part which pronounced my paper "*too severe.*" This member also gave his own judgment of this that it "*was wholly uncalled for.*"

It will be remembered neither of these guilty members, both recently, and, so far as we know, both now, members of the same faculty, denied the facts to be as we have stated, nor attempted excuse or apology for these, for themselves, or associates.*

It was only my comments on them they denounced—the one as "too severe," the other as "wholly uncalled for." Thus it will be seen they admit the facts of their college neglect and breach of trust to be as we have stated. The one confesses judgment against the faculty by urging that they now cease this neglect and breach, and "*begin* to teach pure Homœopathy," and the other by his ready approval of this urging. Why this from either of these guilty ones, but that each knew the truth of former neglect to teach this?

And now we submit, the facts being admitted as we have stated, no comment on them *can be* "*too severe*" which denounces this long-continued crime. And the crime having been so long continued, the rights of these abused students and the rights of the public which incorporated these false colleges "*called*" in thunder tones for the exposure and denunciation of these faculties which have so long abused the confidence of both. Did this professor believe himself when he declared this exposure and denunciation "uncalled for"? Or can the other, before these admitted facts, imagine any of these, from any source, as more severe than these facts justify?

And now, having the confession of these two professors as to what has *not* been taught in their college, as given above, is it not of interest to know what *has* been given to students as a substitute for this neglected and omitted duty? We have the statement of one of these confessing professors as to their intentions in this business, and his views of means and places where

---

* Since writing this paragraph we have learned this urging member is no longer a member of the faculty with which he had previously sinned. The urging on his part, whatever it may have been with his approver, was purely a matter of an awakened *conscience.* Though this has come rather late, we are glad it has come, and we hope it will do him and his late associates, including this approving one, a great deal of good. Will this urging bring forth in them fruits meet for repentance? *Nous verrons.*

"A good homœopathic education can be obtained. It can be almost any. where, *i. e.*, in almost any homœopathic college. We are physicians *first*, and homœopaths *afterward**; and scientific men all the time."†

There it is—his views and plans—in plain words, which all can understand. "A good homœopathic education can be obtained in almost any homœopathic college." This he gives as his judgment. Then let him answer—Why is it *not*, in any one of them, his own included? He gives a sufficient reason why it is not in his own. "Doctors *first*"—"then homœopaths"—"and scientific men all the time"! He evidently would have it understood that his college is especially zealous in making, or in endeavors to make, *scientific doctors* of their students. He can mean nothing else. Then who shall apologize for or excuse such abortive efforts to this end as have year after year turned out classes of what he considers "scientific" doctors, in whose "scientific" attainments the *science* of therapeutics has no place? It has no place here because it had no place in the instructions given by these professors. And now one of them comes before a county medical society, at this late day, and urges that this wicked omission shall stop; that "pure Homœopathy" shall hereafter be taught even in their own college!

And then, "scientific men all the time" is decidedly good. What can be better? The trouble with this kind of "scientific" teaching is that the science of Homœopathy (without which no man can be a "*scientific*" doctor) is wholly left out. This endeavor to so make scientific doctors is no better than, and only, "scientific" fooling. It is giving to the young men who come

---

* Why "*afterward*"? The reason for the creation of this college was not to "make doctors," but because "other colleges would or could not teach Homœopathy." Then why is this objective of this college existence and that of its faculty deferred to cognates of homœopathic science, and deferred so long that this objective is never reached? They have not taught these cognates better than they were taught by their old school predecessors, though they have given their whole time to them. There has been no pretense before or since the creation of this so-called homœopathic college that these cognates were not taught well enough by old physic.

† If they succeed in making them such adepts in "science" as to equal those of the poet, who could

> a hair divide
> Between the west and northwest side.—*Hudibras*, what does it help to make them homœopathic healers, if they continue to leave out of their teaching, as they confessedly have hitherto, the one science indispensable to this, the science of homœopathic therapeutics? Their attempt to make them such without this science has hitherto been only a miserable failure.

to their college for scientific *bread* only a *stone*, not bread at all.

So, confessedly, by these two professors, "pure Homœopathy" is not taught at all in their college, and hence the "urging" of one in his paper to cease from this crime, which urging is approved by the other. What, then, has been and is taught to students who are there gathered that they may receive this "pure" instruction, now so properly urged? What is the substitute for this, here given to these needy young men, and from what source is it derived and inspired? Here is a specimen given to them in the present session;* it will speak for itself. It is not pretended by the student who wrote this, that it is verbatim, as given by the *professor* (sic !), but it is as nearly so as he could make it from memory, a few days after the lecture of which it made a part. He vouches for the accuracy of the specimen as to the principles it states and advocates.

Gonorrhœa is not due to a specific poison.† The abortive treatment, by Nitrate of Silver, excites an inflammation which, as it subsides, carries off the old trouble.‡ It is never to be used except at the commencement of the disease. A strong injection, twenty grains to the ounce, to be used only once, or one-half grain to the ounce, every two hours, stopping when inflammation and a thick, bloody, purulent discharge are reached. Bi. Chloride of Mercury, one grain to thirty or forty thousand of water, supposing bacteria are present,§ is efficacious if used early—four times daily—causes a subsidence of symptoms. Chloride of Zinc, one to two grains to a pint of water, dries up discharge.

In next inflammatory stage abstain from all injections. May, however, use this one for alleviation of pain: ℞ Ext. Opii, one scruple; glycerine, ℥ i; Aq. Pura, ℥ iii; Zinc Sulph., two grains.

This precious specimen is from one elected to teach in a college created to impart a knowledge of the philosophy and practice of specific medicine, *i. e.*, of Homœopathy. And yet in every line of the above he has made plain his utter ignorance of the first principles of these. First, of its philosophy of disease, this of gonorrhœa, for example. Homœopathic philosophy regards this as the representative of one of the divisions of the fundamental causes of chronic diseases. That it is a poison, or miasm, pervading the whole organism; that the troublesome local manifestation of its presence is only one of its results, and no more the disease itself than is the gathering in the handkerchief the influenza which has made its frequent use a necessity.

---

* February 28th, 1887.

† To what is it due then?

‡ Perhaps this is Homœopathy, but the dose is certainly *rather large*.

§ What is a legitimate ground for this supposition? Is this intended in the interest of making his students *scientific* men?

And yet to this teacher the discharge and disuria are all there is of it. Stop these, and *voila—a cure of old physic.* It is of no consequence to these myopic teachers of a "science," which is no *science*—the results of these appliances for a *cure*, which is no cure—the least important of which is the cursing strictures which afflict this class of suffering sinners. In comparison with these, troublesome as they are, they are only insignificant before the ravagings of the sycosis this injecting practice has shut up in the organism—a ravaging and destruction of organs and functions, which cease only with the end of life, unless met and conquered by the specific means found only in the armamentarium of the "*pure Homœopathy*" this urging professor would have his college *begin* to teach. The philosophy this college was created to teach regards all diseases, except those of mechanical or chemical origin, as the outcome of a dynamic impress of some noxia on the life force of the sufferer, which changes its action on one or more of the functions of his bodily organs, so that the harmony of these is lost, with consequences more or less painful and dangerous, according to the nature of the disturbing noxia. This Professor, in the blindness of old physic, can only see two facts as to that of which he was speaking—discharge of a morbid product and painful micturition. Like old physic, he only sees a *local fact*, characterized by these two elements, discharge and pain. And he teaches to treat these with means which the philosophy his college was created to teach, tells him, only shut up the disease itself in the organism it has invaded for a greater and endless destructive activity, unless conquered by the better means known to the philosophy it was his duty to teach, and to teach his class a knowledge of these better means, and how to use them for better results than can come from any amount or kind of injections, which are ever and only an unscientific resort for the cure of an uncomprehended evil. If this is the outcome of the endeavor of this faculty to "make scientific men," then we submit it is the duty of this faculty, and their *first* duty, themselves to learn, before this problem of healing sicknesses, the meaning of this word "*scientific.*" For now this has no place in their thought, either as to diseases or the appropriate means for their cure.

But here is further, from this same Professor, on this same subject:

"The urethra of one man will be more sensitive than that of another, so you may have to feel round* and work up to the right strength.

---

* But what happens to the patient in the mean time if the agents used in this "feeling round" are too potent? "Feeling round" is good, *i. e.*, good

"Some of the ultra homœopaths consider injections injurious, and would not apply a *poultice* or a mus ard plaster, so afraid are they of metastasis, but I don't believe in having it constantly before me as a bugbear. I believe that injections have done harm, but if the proper injection be applied in the proper manner and at the proper time it will be beneficial.

"If I had nothing left in this world to choose from except internal medications and injections, I would take the injections every time."

[*Applause by the class!*]

Which of the three endeavors of this College was this utterance in the interest of? Doctors "*first.*" It must be he regarded himself in the *first* stage, doctors! For there is neither trace nor savor of Homœopathy nor science of any sort in any word of it, unless it be in the expressed faith "that injections have done harm!" How could it be otherwise? If rightly seen, it will be found they have never done anything else. But he believes, further, "If the proper injection is applied in the proper manner and at the proper time, it will be beneficial." But the truth is, there is no such injection, manner, nor time. The only "proper injection" in the treatment of gonorrhœa is *no injection*, and neither manner nor time can sanctify the abomination, which is wholly false in philosophy and destructive in its results.

Is this the teaching by which they would "make doctors"? If so, what *kind* of doctors does it make? Does this kind of teaching contribute in any degree to the second objective of the alleged policy of this faculty, and on which and only this college was erected? *

Would they "make homœopathists *afterward*" by previous teachings which violate all principles of homœopathic philosophy of disease, or is this to "make their students scientific men all the time" on this pabulum, into which no science, homœopathic or allopathic, in any degree or trace, enters? But if this teaching is in the interest of neither of the three grand objectives of the policy of this college faculty in what interest is it given, and why is it permitted?

Then what are "*ultra* homœopathists"? We do not, perhaps,

---

old physic—none of its adherents can do better. They are always doing this, and, like this Professor, always *doing this in the dark!*

* Two years ago the Dean of this faculty in his commencement speech gave as the *raison d' etre* of this college, that *Homœopathy* could not be taught in any then existing allopathic college, and *therefore* this had been created that a *homœopathic* ed ıcation might there be given to students. Then why is it not? Why, after these years, is it found necessary to urge on homœopathic colleges, this one, of course, included, to *begin* after these years to teach "pure Homœopathy"? Why, instead of this, are students given such twaddle as we have quoted above?

exactly understand to whom this term applies. We know of only one kind, those who are loyally obedient to God's law in their clinical duties, and who never cease to search for the specific remedy for the sicknesses they are called to cure till they have found this and given it. They never resort to injections or other old physic means as a substitute for this, knowing the better results which follow the use of the specific remedy. Such as abandon this for the resorts of old physic from ignorance, incapacity, indolence, or any other cause, are *not* homœopathists at all, whatever they may call themselves or others may call them.*

And whoever teaches classes in a homœopathic college to do this is evidently out of his place, and his labors are wholly subversive of that for which the college was created, *i. e.*, the teaching of Homœopathy, and not for teaching classes the superiority of old physic resorts or of "*old nurses.*" Verily, the urging on our colleges to begin the teaching of this *better*, though so long delayed, was neither untimely nor "*uncalled for.*"

And then we may, perhaps, be permitted to remind this teacher of injections that he misapprehends the objections of those who condemn and reject the use of them—these "ultras" of whom he speaks. It is not a fear of "*metastasis*"—a transference of the disease from one locality to another—but, as we have before remarked, that they shut up this in the organism, where it invades all organs, depraves all functions, and, perhaps, in the end, destroys life itself. That the difficulty of cure of this disease by specific medication—and it can be *cured* by nothing else—is immensely increased by these wholly unscientific resorts this teacher so earnestly recommends. It is not enough for him or others to say they have never *seen* such results follow suppression of gonorrhœal discharge. It is quite possible they never have, and it is as possible if these fearful consequences were before their eyes they would not recognize them as results of their favorite cruelty. Why should they? Gonorrhœa is to them only a discharge of morbid product with painful micturition.†

---

*There is no more propriety in the phrase, "*ultra homœopathists*," than there would be in that of *ultra mathematicians*. "Ultra" obed ence to law is an absurdity the most foolish.

† Just as syphilis was to Ricord, only a sore of a certain kind—a chancre. This healed, and to him there was a perfect *cure*. [*Old physic.*] But there was *no cure* at all, though. As Ricord's observation of his cases ended here, it is more than likely he never knew that such *cured cases* in about two weeks appeared at the Hospital St. Louis to be treated for *secondary syphilis!* This is the testimony of one of the most intelligent young physicians this country has raised. In the pursuit of knowledge of his profession he was in daily

These stopped, and to them the disease is cured. They have no knowledge of it in any other manifestation, so when present it is not recognized. They do not see these disasters, it may be, because when they come on their unfortunate patients they go to others for relief, and it may be to such as this teacher designates as "ultras." Does he mean true homœopaths? It is certain these see more of these than is pleasant, and well they know the extreme difficulty of their cure. Let it be understood that our difficulty is not here, with the liberty of any man to practice injections in treating gonorrhœa, if this is the best he knows, but we do object to classes being taught this in a College said to have been created for teaching pure Homœopathy as a substitute for this noble objective, and more and more earnestly we object to the specific means of the homœopathic armamentarium being held up to disparagement before these classes in comparison with these or any other resorts of old physic.

But this devotee of injections is not the only one in this College created for teaching Homœopathy who casts this aside, and teaches for this the resorts of old physic. This is how he does it:

"The physician who trusts to medicine alone in post-partum hemorrhage is culpable. I should feel very bad if any of you should treat it without using the necessary means employed, such as ligature of the limbs with Esmarch's bandage—compression of aorta or femoral arteries—application of ice to interior of the uterus, or hot water and ice alternately—curette the uterus—use application of Iodine, which of itself is a styptic, etc."

Here it is. Could old physic have done better, or any recreant homœopath have done worse? This is given to his class as a substitute for that for which he had been called to the chair he occupies to teach, *viz.: specific* medicine. We venture to say to this man and his class, if he will but teach them this they will have rare need of these clumsy appliances of old physic. If he will but acquaint himself with the resources of specific medicine, and practice with them, and teach others to do the same, we venture to assure him he will find few occasions in the results for "bad feeling" when he finds his pupils engaged in

---

attendance on Ricord's clinic and at the St. Louis, where he met these *cured* patients so uniformly that they became objects of expectation, which was seldom disappointed. This famous specialist very likely never knew that the long list of published cures which made his name famous—the patients were not cured at all, but only damned. Healing the chancre was no more a *cure* than is the drying up the discharge of a gonorrhœa. And who does not know, who knows anything at all of the matter, how immensely the difficulty of the real cure is increased by resorts which produce these deceiving results?

obeying God's law of therapeutics. And more—in a practice of more than forty-five years with the specific medicine we have not met a case which called for any of the fuss and nonsense* he says he will feel bad if the members of his class do not adopt in their practice, to the exclusion of specific medicine! Thus he teaches his class to avoid that for a knowledge of which his College was created, and to teach which he has been placed in the official chair he occupies! Was the crime in the appointment of an untaught, and therefore incompetent, man to this chair, or is it in his willful neglect and perversion of duty? Is there no power in the authority which made this appointment to remedy this abuse of confidence and dereliction of duty?

We have confession of this faculty as to what is *not* taught, and we have seen specimens of what *is*. We have, further, this testimony from a graduate of this college, of two years' standing, as to the spirit in which this, which is not Homœopathy, is taught by this recreant faculty, and also as to the spirit in which it is received by their deceived and misled students:

> "When there was mention of any of the characteristics of true Homœopathy, or any allusions to these made, it was only to hold them up to ridicule! —And this brought from the class their loudest applause, every time!"

The witness to this shameful and disgraceful state of things is intelligent and worthy of belief as to the truth of that which he affirms. He has been, during the two years since his graduation, industriously engaged in obtaining, by his own efforts, that for which he had paid this faculty to impart to him, and at each step in advance in this knowledge he finds new sense of the crime committed against him and his associates by this faculty, created to teach the Homœopathy of Hahnemann, but who, disregarding this duty, only employed themselves in ridiculing it! And yet, a member of this faculty, so engaged, thinks a homœopathic education "can be had in almost any college," and that rebuke of this abominable conduct is "uncalled for"! Who can hereafter, with these statements before him, respect the truth or the judgment of this guilty Professor?

---

* We so characterize these as viewed from the homœopathic standpoint, and in contrast of these with the superior powers of specific medicines, always at the command of the specific prescriber. From the allopathic standpoint, apparently occupied by this teacher, the case must appear different, as these. no doubt, are the best means it knows!

